# Supplementary material for: An analysis of the impact of administrative approval reform on the technological complexity of manufacturing exports
Source: PLoS One. 2025 Apr 25;20(4):e0319734. doi: 10.1371/journal.pone.0319734 (PMC12026959; doi:10.1371/journal.pone.0319734)
Supplement: Attachment — Submitted filename: Response to Reviewers.docx [file pone.0319734.s001.docx]

**An analysis of the impact of administrative approval reform on the technological complexity of manufacturing exports**

We sincerely appreciate the editor's very kind and careful examination of our paper. The reviewer's comments are very right and helpful to us. We are very grateful to the reviewer for their hard work and the important comments for improving the quality of our article.

We have studied all the comments and made revisions very carefully. The following is an outline of the revisions that we have made. (*The blue mark represents the reviewer's question, and the red mark represents the response.*)

**Response to Reviewer 1**

1. The practical value of this research work should be clarified and highlighted in the Abstract, which can help readers understand the engineering background of this research work.

*1. We are very grateful to the reviewer for their suggestions and related reviews. Based on the reviewer's suggestions, we have revised and improved the abstract as follows:*

*As the global economic landscape continues to evolve, the low technological content and persistent lack of international competitiveness in China's manufacturing exports have become increasingly evident, highlighting the urgent need for a shift from "quantity" to "quality" in the sector. Administrative approval reform, a crucial pillar of institutional innovation in the new era, plays a significant role in enhancing the technological complexity of manufacturing exports and strengthening international competitiveness. Using data from 2001 to 2013, this study examines the impact of administrative approval reform on the technological complexity of manufacturing exports, alongside its underlying mechanisms, from both theoretical and empirical perspectives. Theoretical model analysis indicates that the reform effectively raises technological complexity by lowering the marginal and fixed costs associated with adjusting product complexity. Empirical findings confirm that administrative approval reform significantly improves technological complexity, with results remaining robust across various tests. At the micro level, the reduction of institutional transaction costs emerges as a critical channel for the effectiveness of reform. Meanwhile, increasing research and development investment, fixed asset investment, and carrying out technological innovation are key pathways that influence technological complexity. The reform's impact is particularly pronounced for non-state-owned enterprises and firms in coastal and port cities, as shown by a heterogeneity analysis. Additionally, a DOP decomposition of city-level export technological complexity reveals that enhanced inter-firm resource allocation—shifting market share from firms with lower to higher technological complexity—serves as the primary mechanism driving improvements at the city level.*

2. The authors did a good literature review on the current research progress. It is suggested that the research gaps be summarized before presenting and introducing this paper's main contributions and novelty.

*2. We greatly appreciate the reviewer's attention to our work and the constructive suggestions provided. This comment is vital for readers to grasp our research question and objectives in this article. Specifically, as follows (****The specific content can be found in the last paragraph of the fourth page of the text****):*

*Overall, existing research on the relationship between administrative approval reform and the technological complexity of manufacturing exports remains open to further investigation, especially in terms of direct analysis and quantitative assessment of how such reforms facilitate improvements in export technological complexity. Although previous studies have partially elucidated the link between institutional quality and the technological complexity of manufacturing exports, offering valuable theoretical perspectives on the influence of administrative approval reform on export product quality, the specific mechanisms underlying this relationship require more comprehensive and in-depth examination....*

3. It is suggested that the resolutions of the figures in the current manuscript be improved.

*3.Thank you for your valuable feedback. I have carefully considered the issue of digital resolution you raised. Due to limitations in existing software technology, I am unable to provide higher resolution charts, but I have made every effort to optimize the quality of the existing charts to ensure they are more clear in visualization.*

4. There are some grammar errors in this manuscript. Please check the whole manuscript and address these kinds of issues throughout it.

*4. We sincerely appreciate your feedback regarding the grammatical errors in our manuscript. We have thoroughly reviewed the entire document and made the necessary corrections to address these issues throughout. We are committed to ensuring that the manuscript meets the highest standards of clarity and professionalism. Thank you for bringing this to our attention.*

5. It is suggested that some recommendations for future work be included at the end of the Conclusion.

*5. We appreciate the reviewer’s suggestion to include recommendations for future work at the end of the Conclusion. In light of this, we have added the following content:*

*This paper continues to face certain limitations. On the one hand, due to the constraints imposed by the available data, this paper relies on data from 2001 to 2013, which limits its capacity to conduct a long-term dynamic analysis of the impact of administrative approval reform on the technological complexity of manufacturing exports. On the other hand, this research is restricted to exploring the impact of the administrative approval system on the technological complexity of exports. In the context of economic globalization, analyzing the role of institutional optimization in strengthening positions within global value chains represents another fruitful direction for future research. So, future research could focus on two primary aspects: First, leveraging long-term data using regression discontinuity design (RDD) or dynamic panel data models to explore the long-term dynamic effects of administrative approval reform on the evolution of technological complexity in manufacturing exports. Second, applying difference-in-differences (DID) methods to examine the interplay between administrative approval reform and global value chain restructuring, while assessing its varying impacts on export technological complexity across different segments, and clarifying the role of institutional optimization in advancing global value chain positions.*

**Response to Reviewer 2**

1. First, there are a few typographical errors that need to be addressed to improve readability. For instance, on page 4, paragraph 3, line 10, some Chinese characters appear and should be removed. Moreover, on page 6, the subtitle 'Production sector' is repeated and should be corrected. I also recommend reviewing the formatting of headers, titles, and references to ensure full compliance with the journal's guidelines.

*1.* *We greatly appreciate the reviewer's comments. We have carefully reviewed the entire article and made revisions to some low-level errors.*

2. Additionally, there are sections where further clarification would enhance the reader's understanding. In Table 1 on page 11, a more detailed explanation of the data is needed. Specifically, it would be helpful to clearly define each variable in the table and provide more comprehensive information about the data being used.

*2.We greatly appreciate the reviewer's suggestions. Based on the reviewer's suggestions, we have provided a detailed explanation of the relevant variables. Specifically, as follows (****The specific content can be found on page 11 of the article****):*

*(3) Mediating variable.*

*According to theoretical analysis, institutional transaction costs, per capita institutional transaction costs, R&D investment, fixed asset investment, and technological innovation are key mechanisms through which administrative approval reform affects the technological complexity of manufacturing exports.*

*Institutional transaction costs (ITC) and per capita institutional transaction costs (PITC): The reduction in institutional transaction costs is the most direct outcome of administrative approval reform. For measuring these costs, this study follows the approach of Guo and Shao (2019), utilizing the sum of management, financial, and sales expenses, as well as the average of these costs per employee, to assess institutional transaction costs and per capita institutional transaction costs.*

*Fixed asset investment (FAI): Innovation activities and improvements in operational efficiency depend on continuous R&D efforts and fixed asset investment. Fixed asset investment is calculated as the total fixed assets for the current year minus the total fixed assets of the previous year, plus depreciation for the current year.*

*R&D investment (R&D): Measured by the logarithm of a company’s R&D expenditure. Generally, higher R&D investments promote innovation, which in turn enhances the technological complexity of a company’s exports.*

*Technological innovation (INV): Based on previous research, technological innovation is measured by the logarithm of new product output value. A higher value indicates more active innovation efforts, which positively impacts the technological complexity of exported products.*

*(4)Control variables. In this study, we selected the following control variables to ensure a more comprehensive and accurate analysis of the impact of administrative approval system reform on the technological complexity of manufacturing exports:Firm age (Age): Reflects the number of years since the company was established and is measured by the logarithm of the company's age. Older companies may have accumulated more experience and resources, which can influence their capacity for technological innovation and the complexity of their exported products.*

*Industry structure (Sec): Represented by the proportions of secondary, and tertiary industries. The technological requirements vary significantly across these sectors, and optimizing the industry structure, particularly by increasing the share of high-tech industries, can enhance the technological complexity of exported products.*

*Firm size (Scale): Measured by the logarithm of highway freight volume, improved transportation conditions help companies access international markets more quickly, thereby enhancing the technological sophistication of their exported products.*

*Transportation convenience (Commdity): Assessed by highway freight volume. Improved transportation conditions facilitate faster access to international markets and raise the technological sophistication of exported goods.*

*Debt-to-asset ratio (Debt): A key indicator of financial health. Excessive debt may limit a company’s ability to invest in technology, thus impacting the complexity of its exported products.*

*Wage Level (Sarsy): Influences labor costs and the company's ability to attract high-quality talent. Higher wage levels may enable firms to recruit more innovative personnel, thereby enhancing technological complexity.*

*Population density (Peo): Measured by the ratio of urban population to urban area. Higher population density often indicates greater availability of resources and larger market opportunities, which can stimulate technological innovation and complexity.*

*These control variables provide a more detailed perspective for analyzing the impact of administrative approval system reforms on the technological complexity of manufacturing exports, helping to reveal the true effects of these reforms.*

*Table 1 Descriptive statistics*

| *Variable* | *Variable Description* | | *Obs* | *Mean* | *Std.Dev.* | *Min* | *Max* |
| --- | --- | --- | --- | --- | --- | --- | --- |
| *lfirmprody* | *Technological Complexity* | | *668000* | *5.705* | *0.72* | *1.469* | *7.568* |
| *alc_dum* | *Administrative Approval Reform* | | *682000* | *0.71* | *0.454* | *0* | *1* |
| *age* | *Firm Age* | | *682000* | *2.12* | *0.658* | *0* | *7.607* |
| *scale* | *Firm Size* | | *679000* | *5.378* | *1.125* | *2.079* | *12.316* |
| *debt* | *Debt-to-Asset Ratio* | | *606000* | *0.45* | *0.311* | *0* | *1* |
| *sec* | *Industrial Structure* | | *682000* | *51.172* | *8.182* | *8.05* | *92.3* |
| *peo* | *Population Density* | | *678000* | *1.285* | *0.683* | *0.196* | *9.542* |
| *sarsy* | *Wage Level* | | *677000* | *14.291* | *1.448* | *9.274* | *17.976* |
| *commdity* | *Transportation Accessibility* | | *682000* | *9.23* | *0.839* | *2.303* | *13.225* |
| ***ITC*** | | *Institutional transaction costs* | *445000* | *8.536* | *1.466* | *0* | *18.322* |
| ***PITC*** | | *per capita institutional transaction costs* | *443000* | *3.197* | *1.08* | *0* | *12.43* |
| ***R&D*** | | *Fixed asset investment* | *107000* | *0.937* | *2.345* | *0* | *15.782* |
| ***FAI*** | | *R&D investment* | *197000* | *7.968* | *1.955* | *0* | *17.139* |
| *INV* | | *Technological innovation* | *271000* | *1.058* | *3.121* | *0* | *18.015* |

3. It would be beneficial to provide a more detailed explanation of the results from the parallel trends test. For Figure 3, please include a title for the y-axis to clarify the variable being represented. Additionally, further elaboration on the results would be helpful, particularly regarding the observed lag in the first year following the implementation of the administrative approval reform. Similarly, the decrease in year 4 wants more explanation.

*3.* *We greatly appreciate the questions raised by the reviewer. Based on the critical suggestions of the reviewers, we have added relevant content. For Figure 3, we have added a title for the y-axis (as shown below).*

*Figure 3 Parallel trend test*

*In addition, regarding the explanations for the first and fourth years after the implementation of administrative approval reform, the details are as follows (****The specific content can be found on page 15 of the article****):*

*Additionally, We also found, the impact of administrative approval reform on the technological complexity of manufacturing exports demonstrates a lag, with a significant positive effect observed starting from the second year after the establishment of administrative approval centers. The lack of significance of the estimated coefficient in the first year may be attributed to the time required for enterprises to adjust to the new policy environment. During the initial stages of the reform, enterprises need time to adapt to the new policy environment and approval processes. Although the administrative approval reform aims to reduce institutional transaction costs, during its early implementation, enterprises may still need to acclimate to new operational procedures, administrative processes, and regulatory requirements. This adaptation period may lead to a temporary decline in export efficiency, thus affecting the complexity of export technologies. Furthermore, as illustrated in Figure 3, the estimated coefficients begin to decline from the fourth year, indicating a weakening of the reform's positive effects on the technological complexity of manufacturing exports. This trend may arise as enterprises gradually adapt and fully capitalize on the policy benefits, leading to diminishing incremental gains and a plateau in the reform's impact. After several years of adjustment, the marginal benefits of the policy gradually diminish, contributing to the observed decrease in export complexity during the fourth year.*

**Response to Reviewer 3**

1. Expansion of Temporal Analysis: Extending the analysis to include data beyond 2006 could provide insights into the ongoing impact of administrative approval reforms on manufacturing exports, especially in the context of China's rapid economic changes.

*1.* *We greatly appreciate the suggestions and comments from the reviewers. Based on the suggestions of the reviewers, we have updated the data and conducted empirical testing again.* *Since the data used in this study is sourced from the China Industrial Enterprise Database, which has only been updated until 2013, the expanded dataset in this paper is also limited to 2013.*

2. In-depth Treatment of Confounding Variables: Developing a more detailed framework to account for and integrate the impacts from other concurrent policy shifts (e.g., foreign trade agreements, tariff changes) could strengthen the causal inference of the findings.

*2. We greatly appreciate the reviewer's suggestions. Based on the reviewer's suggestion, we have added the impact of relevant policy variables in the article. Specifically, as follows (****The specific content can be found on page 17 of the article****):*

*From 2001 to 2013, China implemented several significant policies, including the reform of state-owned enterprises and accession to the WTO, in addition to administrative approval reform. The timing of these policies closely coincided with that of the administrative approval reform, potentially resulting in estimation biases. To address the potential issue of omitted variables, we separately used the regional proportion of state-owned enterprises and the dependence on foreign trade to measure the reform of state-owned enterprises and the impact of WTO accession, respectively. The results are presented in Table 4. Columns (1) to (3) present the regression results while controlling for other major policies. Regardless of the time dimension or the intensity of the administrative approval reform, it continues to effectively enhance the technological complexity of manufacturing exports. The results remain robust.*

Table 4 Robustness test: Other policy shocks and sample selection bias

| VARIABLES | (1) | (2) | (3) | (4) | (5) | (6) |
| --- | --- | --- | --- | --- | --- | --- |
|  | lfirmprody | lfirmprody | lfirmprody | lfirmprody | lfirmprody | lfirmprody |
|  |  |  |  |  |  |  |
| alc_dum | 0.0035*** |  |  | 0.0035*** |  |  |
|  | (0.0011) |  |  | (0.0011) |  |  |
| alc_department |  | 0.0125*** |  |  | 0.0125*** |  |
|  |  | (0.0042) |  |  | (0.0042) |  |
| alc_item |  |  | 0.0080*** |  |  | 0.0080*** |
|  |  |  | (0.0008) |  |  | (0.0008) |
| IMR |  |  |  | -0.0002 | -0.0002 | -0.0020 |
|  |  |  |  | (0.0013) | (0.0013) | (0.0013) |
| the reform of state-owned enterprises | -0.0453*** | -0.0451*** | -0.1455*** | -0.0452*** | -0.0450*** | -0.1455*** |
|  | (0.0087) | (0.0087) | (0.0078) | (0.0087) | (0.0087) | (0.0078) |
| trade dependence | 0.0773*** | 0.0772*** | 0.2508*** | 0.0774*** | 0.0772*** | 0.2508*** |
|  | (0.0104) | (0.0104) | (0.0099) | (0.0104) | (0.0104) | (0.0099) |
| Constant | 5.6881*** | 5.6892*** | 4.5050*** | 5.6881*** | 5.6891*** | 4.5032*** |
|  | (0.0636) | (0.0637) | (0.0439) | (0.0636) | (0.0637) | (0.0440) |
| Controls | YES | YES | YES | YES | YES | YES |
| Year FE | YES | YES | YES | YES | YES | YES |
| Firm FE | YES | YES | YES | YES | YES | YES |
| Observations | 542,650 | 542,650 | 542,650 | 542,650 | 542,650 | 542,650 |
| R² | 0.7793 | 0.7793 | 0.7714 | 0.7793 | 0.7793 | 0.7714 |

3. Qualitative Insights: Incorporating qualitative data through interviews or case studies of specific enterprises could enrich the analysis and address how administrative reforms have been experienced by businesses firsthand, adding depth to the quantitative findings.

*3. We greatly appreciate the suggestions and comments from the reviewers. Your suggestion is very helpful for our research.* *Based on your suggestion, we included a qualitative analysis in the section "2. Theoretical Analysis" regarding the simplification of approval processes and the reduction of enterprise burdens following administrative approval reforms for specific enterprises. However, as this paper is primarily an empirical study, the in-depth qualitative analysis of administrative approval reform that you suggested will be a priority and a key focus of our future research. Specifically, as follows:*

*We have collected three cases of enterprises benefiting from Guangxi's "full-chain approval service" for entrepreneurship. Case 1: Guangxi Changhong Pharmaceutical Co., Ltd. reduced its required submission materials from 26 items to 23 items, and the processing time was shortened from 35 working days to 10 working days, while the number of in-person visits dropped from 3 to 1. Case 2 and 3: Both Guangxi Chunjiang Food Co., Ltd. and Guangxi Huxin Import & Export Co., Ltd. saw their submission materials decrease from 14 items to 12, processing times reduced from 35 to 10 working days, and the number of visits reduced from 2 to 1. Overall, these enterprises experienced significant simplification of their approval processes, with an average reduction of 25 working days in processing time, along with a notable decrease in submission requirements and in-person visits. This led to a substantial increase in administrative efficiency, alleviating the burden on enterprises and facilitating their rapid growth. Additionally, Liang Pinghan et al. (2020), through a questionnaire survey on the paperless reform of the export tax rebate policy, further confirmed the positive impact of administrative approval reforms on manufacturing enterprises.*

4. Further Exploration of Mechanisms: More specific exploration of the mechanisms by which the administrative approval reform is acting (beyond resource allocation effects) could provide valuable avenues for further research, perhaps utilizing qualitative methodologies as mentioned above.

*4. We greatly appreciate the suggestions and comments from the reviewers. Based on the suggestions of the reviewers, we also conducted a mechanism analysis at the corporate level and found that reducing institutional costs and promoting corporate innovation also played an important role. The specific empirical test is as follows (****The specific content can be found in section 5.2****):*

*Reducing institutional transaction costs is the most immediate effect of administrative approval reform. This section investigates the impact of such reform on institutional transaction costs. Adopting the methodology of Guo and Shao (2019)[23], this study measures institutional transaction costs as the sum of management expenses, financial expenses, and selling expenses, alongside their per capita values to assess per capita institutional transaction costs. The regression outcomes are detailed in Table 8 where Columns (1) and (2) present the effects of administrative approval reform on overall and per capita institutional transaction costs. The estimated coefficients of the key explanatory variable, alc_dum, are negative and statistically significant at the 10% level, suggesting that administrative approval reform effectively reduces institutional transaction costs. Specifically, compared to cities without such reforms, cities implementing these reforms see a 2.14% decrease in institutional transaction costs and a 1.94% reduction in per capita costs.*

*……Therefore, this study will next examine the impact of administrative approval reforms on innovation. Given that successful innovation and improved operational efficiency depend on sustained investment in R&D and fixed assets, this section further investigates whether administrative approval reform fosters increased R&D expenditure and fixed asset investment, and assesses its impact on firms' technological innovation. Fixed asset investment is calculated as the difference between the total fixed assets of the current year and the previous year, plus the current year’s depreciation. Technological innovation is measured by the output value of new products. Columns (3) to (5) of Table 8 present the estimated effects of administrative approval reform on R&D expenditure, fixed asset investment, and technological innovation. The coefficients of alc_dum are positive and statistically significant at the 1% level across all metrics.*

*Table 8 Mechanism verification*

| *VARIABLES* | *(1)* | *(2)* | *(3)* | *(4)* | *(5)* |
| --- | --- | --- | --- | --- | --- |
|  | ***ITC*** | ***PITC*** | ***R&D*** | ***FAI*** | *INV* |
|  |  |  |  |  |  |
| *alc_dum* | *-0.0214*** | *-0.0194** | *0.0568**** | *0.0256**** | *0.0699**** |
|  | *(0.0093)* | *(0.0107)* | *(0.0054)* | *(0.0050)* | *(0.0052)* |
| *Constant* | *6.6270**** | *6.3105**** | *-1.2307**** | *6.3728**** | *0.8047**** |
|  | *(0.2126)* | *(0.3161)* | *(0.1639)* | *(0.3060)* | *(0.1787)* |
| *Controls* | *YES* | *YES* | *YES* | *YES* | *YES* |
| *Year FE* | *YES* | *YES* | *YES* | *YES* | *YES* |
| *Firm FE* | *YES* | *YES* | *YES* | *YES* | *YES* |
| *Observations* | *477,134* | *477,134* | *104,948* | *166,158* | *267,236* |
| *R²* | *0.9178* | *0.8925* | *0.6763* | *0.7833* | *0.4789* |

5. Comparison with Other Regions: A comparative analysis with other countries unde rgoing similar reforms could draw informative parallels and enhance the understanding of the broader implications of administrative reform on manufacturing export complexity.

*5.Thank you for your valuable suggestion. In response to your comment, we have made the necessary additions to the paper by analyzing the experiences of other countries that have undergone similar administrative reforms. Specifically, as follows (****The specific content can be found in the last paragraph of page 21 of the article)****:*

*the United States, the European Union, and the OECD have all experienced reforms in their regulatory systems. In the U.S., reforms were implemented through the establishment of a robust legal framework, the issuance of executive orders, and the creation of regulatory oversight bodies such as the Office of Management and Budget (OMB) and the Office of Information and Regulatory Affairs (OIRA). These measures effectively streamlined government regulatory processes, reduced institutional transaction costs, and improved market efficiency. The EU, on the other hand, standardized regulatory practices across member states through the Impact Assessment Guidelines, which primarily address market and regulatory failures. This effort enhanced regulatory consistency and transparency, promoting better resource allocation and increasing economic competitiveness. Similarly, the OECD improved regulatory efficiency by simplifying administrative procedures and introducing regulatory impact analysis tools such as cost-benefit analyses and risk assessments, thus enhancing the scientific and effective nature of policy decision-making.*

*These experiences demonstrate that administrative approval reforms play a crucial role not only in enhancing government efficiency and transparency but also in optimizing resource allocation, reducing unnecessary institutional costs, and fostering innovation—thereby driving economic growth and market vitality. Drawing from these countries' experiences, we gain a broader understanding of the potential impact of administrative approval reforms on the technological complexity of manufacturing exports, particularly in promoting technological innovation, increasing export complexity, and enhancing international competitiveness.*

*We believe these additions further enrich the analys.*
