# Supplementary material for: An analysis of the impact of administrative approval reform on the technological complexity of manufacturing exports
Source: PLoS One. 2025 Apr 25;20(4):e0319734. doi: 10.1371/journal.pone.0319734 (PMC12026959; doi:10.1371/journal.pone.0319734)
Supplement: Attachment — Submitted filename: Response to Reviewers (1)(1).docx [file pone.0319734.s002.docx]

**An analysis of the impact of administrative approval reform on the technological complexity of manufacturing exports**

We sincerely appreciate the editor's very kind and careful examination of our paper. The reviewer's comments are very right and helpful to us. We are very grateful to the reviewer for their hard work and the important comments for improving the quality of our article.

We have studied all the comments and made revisions very carefully. The following is an outline of the revisions that we have made. (*The blue mark represents the reviewer's question, and the red mark represents the response.*)

**Response to Reviewer 2**

1. Checking the whole manuscript and addressing all grammatical errors is still recommended.

*1.Thank you for your valuable feedback and suggestion. We greatly appreciate your attention to detail regarding the grammatical accuracy of the manuscript. Following your recommendation, we have conducted a thorough review of the entire manuscript and addressed all identified grammatical errors to ensure clarity and precision.*

**Response to Reviewer 3**

1. Expand Sample Size: Future research should consider a larger and more diverse sample to enhance the validity and applicability of the findings.

*1.* *We sincerely appreciate your valuable suggestion regarding expanding the sample size, and we fully understand the critical role that a larger and more diverse sample plays in enhancing the validity and applicability of research findings. In this empirical study, our analysis requires detailed financial data from manufacturing firms. Based on a review of existing studies and the available data sources, the China Industrial Enterprise Database published by the National Bureau of Statistics is currently the only dataset in China that offers both a relatively large sample size and comprehensive financial data for manufacturing firms. Therefore, due to the limitations of data availability, this study has utilized the largest accessible sample size to conduct an in-depth analysis.*

*Nonetheless, we recognize the importance of expanding data sources and will continue to closely monitor new data developments. This will enable us to further optimize sample size and structure in future research, thereby improving the quality and robustness of our studies.*

2. Incorporate Longitudinal Approaches: Long-term data collection could improve the understanding of trends and causal relationships.

*2.The data used in this study comes from the China Industrial Enterprise Database, published by the National Bureau of Statistics. To the best of our knowledge, the database has only been updated to 2015. However, due to concerns about data reliability, most scholars currently utilize data up to 2013. For example, articles focusing on topics related to Chinese manufacturing firms generally rely on data from 2013 or earlier. When studying manufacturing firm productivity, researchers often use data from as early as 2007 or before.*

*Examples include:*

*Si and Luo (2024), in Journal of Asian Economics, "The productivity spillover effect of foreign divestment: Evidence from Chinese industrial enterprises" (https://doi.org/10.1016/j.asieco.2024.101824), utilized data from 1998–2007.*

*Zhang et al. (2024), in International Review of Economics & Finance, "The Government's fiscal and taxation policy effect on enterprise productivity: Policy choice and optimal allocation" (https://doi.org/10.1016/j.iref.2024.03.049), used data from 1998–2012.*

*Zhang et al. (2024), in Journal of Asian Economics, "Can development zones reduce energy consumption and carbon emissions of enterprises? Evidence from China" (https://doi.org/10.1016/j.asieco.2024.101845), used data from 2000–2013.*

*He et al. (2024), in Economic Analysis and Policy, "Industrial robots and pollution: Evidence from Chinese enterprises" (https://doi.org/10.1016/j.eap.2024.03.001), relied on data from 1998–2013.*

*Cao et al. (2025), in Technological Forecasting and Social Change, "Robot adoption and firm export: Evidence from China" (https://doi.org/10.1016/j.techfore.2024.123878), used data from 2011–2013.*

3. Enhance Visual Aids: Improving the clarity and presentation of visual data could help in conveying complex results more effectively.

*3.Thank you very much for your valuable suggestions. As our empirical analysis was conducted using Stata, all the figures were generated by the software, and unfortunately, we are unable to enhance the clarity of the data visualizations in this study. We will keep your recommendations in mind for future research and ensure the use of software capable of producing higher-quality visualizations.*

4. Broaden Literature Review: The authors should aim to include a wider range of studies in their literature review to provide a richer context for their findings.

*4.Thank you for your valuable suggestions. In response, we have thoroughly reorganized the literature review as per your recommendations and presented it as a standalone chapter, now positioned as Chapter 2, titled "Literature Review." The specific revisions are as follows:*

*2. Literature review*

*The administrative approval system reform, a critical mechanism for reshaping the business environment, has garnered significant attention from both the economic research community and policymakers in recent years. Zhu and Zhang (2016) were pioneers in exploring the diffusion process of China’s administrative approval system reform, analyzing the determinants influencing local governments’ decisions to establish administrative approval centers[1]. Building on this foundation, subsequent research has delved into the economic benefits derived from the reform from various perspectives. Xia and Liu (2017), through robust empirical analysis, identified a significant positive correlation between administrative approval system reform and overall economic growth[2]. By streamlining approval procedures and reducing administrative interference, the reform has effectively increased the participation of market entities and improved resource allocation efficiency, injecting new momentum into macroeconomic growth. Liu and Zhong (2018) further highlighted the pivotal role of administrative approval reform in addressing overcapacity issues[3]. Under the traditional administrative approval framework, local governments, driven by factors such as promotion incentives, often overexercised their approval powers to facilitate capacity expansion. The reform, by curbing the improper exercise of such powers, has guided capacity structures toward a more rational, efficient, and sustainable optimization pathway, thereby promoting the structural adjustment and upgrading of the broader economy. Lai et al. (2018) underscored the critical role of administrative approval system reform in optimizing market resource allocation[4]. The reform reduces the dispersion in productivity distribution across cities, facilitates a more balanced and efficient flow of resources across regions and industries, and ultimately enhances the operational efficiency and stability of the economic system.*

*At the enterprise level, the administrative approval system reform has also instigated profound and transformative impacts. Wang and Feng (2018) employed the difference-in-differences model to test and found that the administrative approval system reform has remarkably promoted the elevation of enterprise innovation levels and the optimization of production efficiency[5]. After the reform, the simplification and optimization of the approval process have substantially reduced the time costs and energy expenditures of enterprises in key operational aspects such as project approval and qualification acquisition, enabling enterprises to allocate more resources to core competitiveness-building areas like R & D innovation and production process improvement, thereby vigorously enhancing the core competitiveness of enterprises in market competition. Chen (2015) found that the decline in regional administrative approval intensity can significantly lower the cost threshold for enterprises to initiate entrepreneurial activities and reduce the institutional barriers to enterprise access[6]. This transformation has created a more relaxed and favorable institutional environment for the birth and growth of emerging enterprises, significantly increasing the probability of enterprises entering the market and effectively promoting the formation of a diversified pattern of market entities and the intensification of market competition. Zhang et al. (2016) further discovered that the administrative approval system reform not only affects the enterprise establishment stage but also can remarkably augment the individual entrepreneurship probability and expand the local entrepreneurship scale, comprehensively stimulating the entrepreneurial vitality and innovation spirit of the whole society and cultivating more new growth points for macroeconomic development[7]. Additionally, Cheng et al. (2023) demonstrated that the optimization of the administrative approval process can directly stimulate a significant increase in enterprise investment levels[8]. The administrative approval system reform, by enhancing government administrative efficiency, effectively reduces the information cost obstacles faced by enterprises in the investment decision-making process, augments the flexibility and precision of investment decisions, and renders enterprises more proactive and scientific in resource allocation decision-making. Zheng et al. (2023) and Zhu et al.(2021) further corroborated that the administrative approval reform has a significant promoting effect on the improvement of enterprise productivity. Although certain differences may exist among different industries, regions, and enterprise ownership types, in general, the reform has laid a solid foundation for the enhancement of enterprise comprehensive competitiveness through multiple channels such as optimizing resource allocation efficiency, promoting the diffusion of technological innovation, and improving enterprise internal management efficiency[9][10].*

*In the field of international trade research, the significance of the institutional environment and business environment has also been widely and deeply recognized. In 1990, North pioneered the classic proposition of "institutions initiate trade", highlighting the fundamental and leading role of institutions in the international trade system. Since then, numerous scholars have further delved into the theoretical connotations and empirical evidences of the specific impact mechanisms of institutional quality on international trade. It has been found that institutional comparative advantage has become one of the key factors determining the choice of international trade modes and the pattern of division of labor. Differences in institutional arrangements among different countries or regions, such as the degree of perfection of intellectual property protection systems and the stability and predictability of trade policies, profoundly influence their strategic choices of specialized division of labor positioning and trade mode in international trade (Yang, 2019[11]). Dai and Jin (2014)[12], from the perspective of division of labor evolution, found that institutional quality has a significant promoting effect on the technological complexity of a country's export products. Meanwhile, the degree of intra-product international division of labor and its interaction with institutional quality also have a significant promoting effect on the enhancement of export technological complexity. Dai and Zheng (2015) further revealed that there is a close internal relationship between institutional quality and the division of labor position of a country's manufacturing industry in the global value chain[13]. A high-quality institutional environment can effectively attract more high-end production factors, drive the manufacturing industry to ascend and transform towards high value-added links, and thus occupy a favorable strategic position and competitive advantage in the global value chain competition (Hu and Zhang, 2015; Du and Peng, 2019[14][15]). Additionally, Zhang and Yang (2022) found that the optimization of the business environment can improve the position of private enterprises in the value chain[16]. Dai (2020) believed that this positive promoting effect stems not only from the direct effect of business environment optimization but also from the indirect effect formed through intermediary roles such as value chain migration, innovation activity enhancement, and trade condition improvement[17].*

*Specifically regarding the administrative approval system reform, Nunn (2007) was the first to propose that the administrative approval efficiency of a country (region) has a significant impact on domestic economic growth and foreign trade comparative advantage[18]. The level of administrative approval efficiency is directly related to the enterprise's operating cost structure, innovation speed, and market response ability, and thus is manifested as differential performance of comparative advantage in the international trade competition pattern (Feng et al., 2018; Liu and Shen, 2019; Mao and Hu, 2024[19][20][21]). Feng et al. (2018) empirically found that the establishment of administrative approval centers can effectively promote more manufacturing enterprises to enter the international market to participate in competition[19]. Although the effect of policy reform may have a time lag, from a long-term dynamic perspective, it can significantly expand the export scale and market share of cities and industries. On this basis, Liu and Shen (2019) further conclusively confirmed that after China's administrative approval system reform, the division of labor position of manufacturing enterprises in the global value chain has shown a significant upward trend[20]. Mao and Hu (2024) also found that the administrative approval reform has increased the general trade export ratio of enterprises and has a significant promoting effect on the transformation and upgrading of export trade[21]. Alleviating financing constraints, intensifying market competition, and improving enterprise innovation ability are important channels through which the administrative approval reform promotes the transformation and upgrading of export trade. Xu and Wang (2018) took the decentralization of export tax rebate (exemption) approval power in 2006, a specific administrative approval system reform in China, as a quasi-natural experiment and found that the experimental group enterprises have achieved significant improvements in key indicators such as export scale, product quality, and export performance[22].*

*Overall, existing research on the relationship between administrative approval reform and the technological complexity of manufacturing exports remains insufficient, particularly in terms of direct analysis and quantitative assessment of how such reforms contribute to enhancing export technological complexity. Although previous studies have provided partial insights into the link between institutional quality and the technological complexity of manufacturing exports, offering valuable theoretical perspectives on the impact of administrative approval reform, the specific mechanisms underlying this relationship require further comprehensive and in-depth investigation.*

*Compared with existing research, this study makes the following three key contributions: (1) Innovative Research Perspective: This study empirically examines the impact of administrative approval reform on the technological complexity of manufacturing exports using enterprise-level microdata. By addressing the limitations of prior research, it provides a more nuanced understanding of the relationship between administrative reforms and export technological complexity. (2) Theoretical Contribution: Extending the model proposed by Hallak and Sivadasan (2013), this study conceptualizes administrative approval reform as a mechanism for reducing institutional transaction costs[23]. By lowering the marginal and fixed costs associated with adjusting product complexity, the reform influences firms’ decisions on technological complexity. This approach elucidates the underlying mechanisms through which administrative approval reform impacts the technological sophistication of exports. (3) Empirical Insights: The study confirms that administrative approval reform significantly enhances the technological complexity of manufacturing exports. Moreover, it explores the heterogeneous effects of these reforms across cities with varying characteristics, offering a deeper understanding of regional disparities. Additionally, it investigates the micro-level mechanisms underlying the relationship, focusing on the reduction of institutional transaction costs, innovation stimulation, and resource reallocation efficiency.*

*As part of the reorganization of the literature review, we have incorporated additional relevant studies. The newly added references are as follows:*

*Zhu X F., Zhang Y L., Political Mobility and Dynamic Diffusion of Innovation: The Spread of Municipal Pro-Business Administrative Reform in China[J]. Journal of Public Administration Research and Theory,2016, 26(3):535-551.*

*Lai M, Yu Y Z, Liu D Y., Institutional Environment, Government Efficiency and 'Mass Entrepreneurship and Innovation'—Evidence from Cross-Country Experience" [J]. Nankai Economic Studies, 2018, (01): 19-33.*

*Chen G. Regulation and Entrepreneurship—Micro Evidence from China [J]. Management World, 2015, (05): 89-99+187-188.*

*Zhang L. P., Jiang W., Zhou L. Q. A Study on the Impact of Administrative Approval on Entrepreneurship—From the Perspective of Entrepreneurial Ability[J]. China Industrial Economics. 2016 (04): 57-74.*

*Cheng B., Guo Z M., Xiong T., Qiu B Y., Does the administrative approval reform impact firm's investment decisions?[J]. International Review of Economics & Finance, 2023(88): 1052-1067.*

*Zheng X M., Wu C K., Nepal R., Did the administrative approval reform in China affect the productivity of energy firms? – A quasi-natural experimental approach[J]. Energy Economics, 2022(105): 105564.*

*Yang, Q. L. Full Cost, Comparative Advantage and International Trade [M]. Shanghai: Truth & Wisdom Press, Shanghai People's Publishing House. 2019.*

*Dai X., Zheng L. How Does Institutional Quality Affect China's Climbing Up the Global Value Chain? [J]. Journal of International Trade, 2015, (12): 51-63+132.*

*Hu Z. L., Zhang Y. Can the Improvement of Institutional Quality Enhance the Division of Labor Status in the Value Chain? [J]. World Economy Studies, 2015(8): 19-26+127.*

*Du Y. S., Peng D. D. Import Complexity of Producer Services, Institutional Quality and the Division of Labor Status of Manufacturing Industry—Based on the World Input-Output Tables from 2000 to 2014 [J]. Journal of International Trade, 2019(1), 41-53.*

*Zhang W. M., Yang G. Z.The Impact of the Business Environment on the Competitiveness of Private Enterprises [J]. Finance & Trade Economics, 2022, 43(10), 119-133. Quarterly Journal of Economics, 2007, 122(2):569-600.*

*Dai X. Can the Optimization of the Business Environment Improve the Status in the Global Value Chain Division of Labor? [J]. Economic Theory & Economic Management, 2020(5), 48-61.*

*Nunn, N.Relationship-specificity, Incomplete Contracts, and the Pattern of Trade[J].Quarterly Journal of Economics, 2007, 122 (2) :569-600.*

*Feng X., Wang Y. J., Liu, C. L. Administrative Approval Efficiency and China's Manufacturing Exports—A "Quasi-Natural Experiment" Based on the Establishment of Administrative Approval Centers [J]. Journal of Finance and Economics, 2018, 44(10), 98-110.*

*Liu S., Shen M. H. Administrative Approval System Reform and the Division of Labor Status of Manufacturing Enterprises in the Global Value Chain[J]. Reform,2019 (1), 150-158.*
