# Supplementary material for: An analysis of the impact of administrative approval reform on the technological complexity of manufacturing exports
Source: PLoS One. 2025 Apr 25;20(4):e0319734. doi: 10.1371/journal.pone.0319734 (PMC12026959; doi:10.1371/journal.pone.0319734)
Supplement: Attachment — Submitted filename: Response_to_Reviewers_auresp_3.docx [file pone.0319734.s003.docx]

**An analysis of the impact of administrative approval reform on the technological complexity of manufacturing exports**

We sincerely appreciate the editor's very kind and careful examination of our paper. The reviewer's comments are very right and helpful to us. We are very grateful to the reviewer for their hard work and the important comments for improving the quality of our article.

We have studied all the comments and made revisions very carefully. The following is an outline of the revisions that we have made. (*The blue mark represents the reviewer's question, and the red mark represents the response.*)

**Response to Reviewer 3**

**Weaknesses:**

3.Potential Bias from Sample Selection: Although the study appears to address sample selection bias through a Heckman approach, the authors might consider detailing their methodology in this area more clearly to enhan	ce transparency and replicability.

*3.Thank you for the expert's suggestion. We have added the following content to Section 6) Selection Bias on page 20:*

*The data for this study are sourced from the Industrial Enterprise Database and the Customs Database, which include a significant number of domestic sales enterprises within the sample. Excluding these domestic sales enterprises when calculating the technological complexity of manufacturing exports may introduce sample selection bias. To address this issue, we adopt the methodology outlined by Su et al. (2018) [29] and employ the Heckman two-step procedure for robustness checks.*

*First, we use the Probit model to estimate the probability of enterprises engaging in export activities. Specifically, we establish a latent variable model that takes into account various key factors influencing enterprises' export decisions, allowing us to determine whether an enterprise has an export inclination. Based on this, we calculate the Inverse Mills Ratio (IMR). Additionally, to enhance the validity of model estimation, following the recommendation of Su et al. (2018) [29], we ensure that at least one variable from the control variables in the export participation decision equation is excluded from the set of control variables in the equation determining the quality of the enterprise's export products. In line with existing literature, this paper selects the enterprise's lagged one-period export dummy variable as the exclusion variable.*

*We then incorporate the IMR into the key regression equation and perform a re-examination of the original Equation (13). If the estimated coefficient of the IMR is significantly positive, it would indicate the presence of sample selection bias.*

**Areas That Needs Improvements:**

1. Expand Data: Future research should leverage more extensive datasets, preferably incorporating more recent years or longitudinal data, to evaluate trends over time, particularly after significant global events that might influence trade dynamics.

*1.* *We sincerely appreciate your valuable suggestion regarding expanding the sample size, and we fully understand the critical role that a larger and more diverse sample plays in enhancing the validity and applicability of research findings.*

*In this empirical study, our analysis requires detailed financial data from manufacturing enterprises. After reviewing existing research and available data sources, we found that the China Industrial Enterprises Database, published by the National Bureau of Statistics, is currently the only dataset in China that provides comprehensive financial data for manufacturing enterprises with a relatively large sample size. Therefore, due to data availability constraints, this study adopts the largest accessible sample size for in-depth analysis.*

*The data used in this study comes from the China Industrial Enterprises Database, published by the National Bureau of Statistics. This database has only been updated through 2015. Due to concerns about data reliability, most scholars use data up to 2013, and when studying the productivity of manufacturing firms, researchers often rely on data from 2007 or earlier. The following articles are cited as examples:*

*Examples include:*

*Si and Luo (2024), in Journal of Asian Economics, "The productivity spillover effect of foreign divestment: Evidence from Chinese industrial enterprises" (https://doi.org/10.1016/j.asieco.2024.101824), utilized data from 1998–2007.*

*Zhang et al. (2024), in International Review of Economics & Finance, "The Government's fiscal and taxation policy effect on enterprise productivity: Policy choice and optimal allocation" (https://doi.org/10.1016/j.iref.2024.03.049), used data from 1998–2012.*

*Zhang et al. (2024), in Journal of Asian Economics, "Can development zones reduce energy consumption and carbon emissions of enterprises? Evidence from China" (https://doi.org/10.1016/j.asieco.2024.101845), used data from 2000–2013.*

*He et al. (2024), in Economic Analysis and Policy, "Industrial robots and pollution: Evidence from Chinese enterprises" (https://doi.org/10.1016/j.eap.2024.03.001), relied on data from 1998–2013.*

*Cao et al. (2025), in Technological Forecasting and Social Change, "Robot adoption and firm export: Evidence from China" (https://doi.org/10.1016/j.techfore.2024.123878), used data from 2011–2013.*

*Nevertheless, we fully recognize the importance of expanding data sources and will continue to closely monitor emerging data trends. This will enable us to further optimize the sample size and structure in future research, thereby enhancing the quality and robustness of our studies.*

2. Deepen Mechanism Analysis: Expanding on the mechanisms underlying the observed relationships between reforms and technological complexity could offer richer insights. This might include qualitative case studies or interviews with industry stakeholders.

*2. We greatly appreciate the suggestions and comments from the reviewers. Your suggestion is very helpful for our research. Based on your suggestion, we included a qualitative analysis in the section "3. Theoretical Analysis" regarding the simplification of approval processes and the reduction of enterprise burdens following administrative approval reforms for specific enterprises. However, as this paper is primarily an empirical study, the in-depth qualitative analysis of administrative approval reform that you suggested will be a priority and a key focus of our future research. Specifically, as follows:*

*We have collected three cases of enterprises benefiting from Guangxi's "full-chain approval service" for entrepreneurship. Case 1: Guangxi Changhong Pharmaceutical Co., Ltd. reduced its required submission materials from 26 items to 23 items, and the processing time was shortened from 35 working days to 10 working days, while the number of in-person visits dropped from 3 to 1. Case 2 and 3: Both Guangxi Chunjiang Food Co., Ltd. and Guangxi Huxin Import & Export Co., Ltd. saw their submission materials decrease from 14 items to 12, processing times reduced from 35 to 10 working days, and the number of visits reduced from 2 to 1. Overall, these enterprises experienced significant simplification of their approval processes, with an average reduction of 25 working days in processing time, along with a notable decrease in submission requirements and in-person visits. This led to a substantial increase in administrative efficiency, alleviating the burden on enterprises and facilitating their rapid growth. Additionally, Liang Pinghan et al. (2020), through a questionnaire survey on the paperless reform of the export tax rebate policy, further confirmed the positive impact of administrative approval reforms on manufacturing enterprises.*

3. Enhance Methodological Rigor: Addressing potential endogeneity in more innovative ways, perhaps through natural experiments or quasi-experimental designs, could help in establishing stronger causal claims.

*3.Thank you for the expert's suggestion. The method used in our empirical analysis is Difference-in-Differences (DID), and in Section 5.2 "Robustness Test," we conduct a parallel trend test to demonstrate the rigor of the methodology employed in this study.*

4. Broaden International Perspective: Adding an international perspective could enhance understanding. Comparative analyses with other countries that have undergone similar reforms could yield valuable lessons.

*4.Thank you for your valuable suggestion. In response to your comment, we have made the necessary additions to the paper by analyzing the experiences of other countries that have undergone similar administrative reforms. Specifically, as follows (The specific content can be found in the last paragraph of page 24 of the article):*

*the United States, the European Union, and the OECD have all experienced reforms in their regulatory systems. In the U.S., reforms were implemented through the establishment of a robust legal framework, the issuance of executive orders, and the creation of regulatory oversight bodies such as the Office of Management and Budget (OMB) and the Office of Information and Regulatory Affairs (OIRA). These measures effectively streamlined government regulatory processes, reduced institutional transaction costs, and improved market efficiency. The EU, on the other hand, standardized regulatory practices across member states through the Impact Assessment Guidelines, which primarily address market and regulatory failures. This effort enhanced regulatory consistency and transparency, promoting better resource allocation and increasing economic competitiveness. Similarly, the OECD improved regulatory efficiency by simplifying administrative procedures and introducing regulatory impact analysis tools such as cost-benefit analyses and risk assessments, thus enhancing the scientific and effective nature of policy decision-making.*

*These experiences demonstrate that administrative approval reforms play a crucial role not only in enhancing government efficiency and transparency but also in optimizing resource allocation, reducing unnecessary institutional costs, and fostering innovation—thereby driving economic growth and market vitality. Drawing from these countries' experiences, we gain a broader understanding of the potential impact of administrative approval reforms on the technological complexity of manufacturing exports, particularly in promoting technological innovation, increasing export complexity, and enhancing international competitiveness.*

*We believe these additions further enrich the analys.*

5. Improved Data Visualization: Future revisions should consider enhancing figures, charts, and tables used to present findings, ensuring they are accessible and informative. Clearer legends, color-coding, and detailed annotations could help convey complex data more effectively.

*5.Thank you very much for your valuable suggestions. As our empirical analysis was conducted using Stata, all the figures were generated by the software, and unfortunately, we are unable to enhance the clarity of the data visualizations in this study. We will keep your recommendations in mind for future research and ensure the use of software capable of producing higher-quality visualizations.*
